# Semaglutide and diuretic use in obesity-related heart failure with preserved ejection fraction: a pooled analysis of the STEP-HFpEF and STEP-HFpEF-DM trials

**DOI:** 10.1093/eurheartj/ehae322

**Published:** 2024-05-13

**Authors:** Sanjiv J Shah, Kavita Sharma, Barry A Borlaug, Javed Butler, Melanie Davies, Dalane W Kitzman, Mark C Petrie, Subodh Verma, Shachi Patel, Khaja M Chinnakondepalli, Mette N Einfeldt, Thomas J Jensen, Søren Rasmussen, Rabea Asleh, Tuvia Ben-Gal, Mikhail N Kosiborod

**Affiliations:** Division of Cardiology, Department of Medicine, Northwestern University Feinberg School of Medicine, Chicago, IL, USA; Division of Cardiology, The Johns Hopkins University School of Medicine, Baltimore, MD, USA; Department of Cardiovascular Medicine, Mayo Clinic, Rochester, MN, USA; Baylor Scott and White Research Institute, Dallas, TX, USA; Department of Medicine, University of Mississippi, Jackson, MS, USA; Diabetes Research Centre, University of Leicester, Leicester, UK; NIHR Leicester Biomedical Research Centre, Leicester, UK; Department of Internal Medicine, Sections on Cardiovascular Medicine and Geriatrics/Gerontology, Wake Forest University School of Medicine, Winston-Salem, NC, USA; School of Cardiovascular and Metabolic Health, University of Glasgow, Glasgow, UK; Division of Cardiac Surgery, Li Ka Shing Knowledge Institute of St Michael’s Hospital, Unity Health Toronto, University of Toronto, Toronto, ON, Canada; Department of Cardiovascular Disease, Saint Luke’s Mid America Heart Institute, Kansas City, MO, USA; Department of Cardiovascular Disease, Saint Luke’s Mid America Heart Institute, Kansas City, MO, USA; Novo Nordisk A/S, Søborg, Denmark; Novo Nordisk A/S, Søborg, Denmark; Novo Nordisk A/S, Søborg, Denmark; Heart Institute, Hadassah University Medical Center, Faculty of Medicine, Hebrew University of Jerusalem, Jerusalem, Israel; Heart Failure Unit, Cardiology Department, Rabin Medical Center, Faculty of Medicine, Tel Aviv University, Tel Aviv, Israel; Department of Cardiovascular Disease, Saint Luke’s Mid America Heart Institute, Kansas City, MO, USA

**Keywords:** Glucagon-like peptide-1 receptor agonist, Clinical trial, Loop diuretics, Obesity, Heart failure with preserved ejection fraction

## Abstract

**Background and Aims:**

In the STEP-HFpEF trial programme, treatment with semaglutide resulted in multiple beneficial effects in patients with obesity-related heart failure with preserved ejection fraction (HFpEF). Efficacy may vary according to baseline diuretic use, and semaglutide treatment could modify diuretic dose.

**Methods:**

In this pre-specified analysis of pooled data from the STEP-HFpEF and STEP-HFpEF-DM trials (*n* = 1145), which randomized participants with HFpEF and body mass index ≥ 30 kg/m^2^ to once weekly semaglutide 2.4 mg or placebo for 52 weeks, we examined whether efficacy and safety endpoints differed by baseline diuretic use, as well as the effect of semaglutide on loop diuretic use and dose changes over the 52-week treatment period.

**Results:**

At baseline, across no diuretic (*n* = 220), non-loop diuretic only (*n* = 223), and loop diuretic [<40 (*n* = 219), 40 (*n* = 309), and >40 (*n* = 174) mg/day furosemide equivalents] groups, there was progressively higher prevalence of hypertension and atrial fibrillation; and greater severity of obesity and heart failure. Over 52 weeks of treatment, semaglutide had a consistent beneficial effect on change in body weight across diuretic use categories (adjusted mean difference vs. placebo ranged from −8.8% [95% confidence interval (CI) −10.3, −6.3] to −6.9% [95% CI −9.1, −4.7] from no diuretics to the highest loop diuretic dose category; interaction *P* = .39). Kansas City Cardiomyopathy Questionnaire clinical summary score improvement was greater in patients on loop diuretics compared to those not on loop diuretics (adjusted mean difference vs. placebo: +9.3 [6.5; 12.1] vs. +4.7 points [1.3, 8.2]; *P* = .042). Semaglutide had consistent beneficial effects on all secondary efficacy endpoints (including 6 min walk distance) across diuretic subgroups (interaction *P* = .24–.92). Safety also favoured semaglutide vs. placebo across the diuretic subgroups. From baseline to 52 weeks, loop diuretic dose decreased by 17% in the semaglutide group vs. a 2.4% increase in the placebo group (*P* < .0001). Semaglutide (vs. placebo) was more likely to result in loop diuretic dose reduction (odds ratio [OR] 2.67 [95% CI 1.70, 4.18]) and less likely dose increase (OR 0.35 [95% CI 0.23, 0.53]; *P* < .001 for both) from baseline to 52 weeks.

**Conclusions:**

In patients with obesity-related HFpEF, semaglutide improved heart failure-related symptoms and physical limitations across diuretic use subgroups, with more pronounced benefits among patients receiving loop diuretics at baseline. Reductions in weight and improvements in exercise function with semaglutide vs. placebo were consistent in all diuretic use categories. Semaglutide also led to a reduction in loop diuretic use and dose between baseline and 52 weeks.

**Clinical Trial Registration:**

NCT04788511 and NCT04916470.

## Structured Graphical Abstract

**Graphical Abstract:**
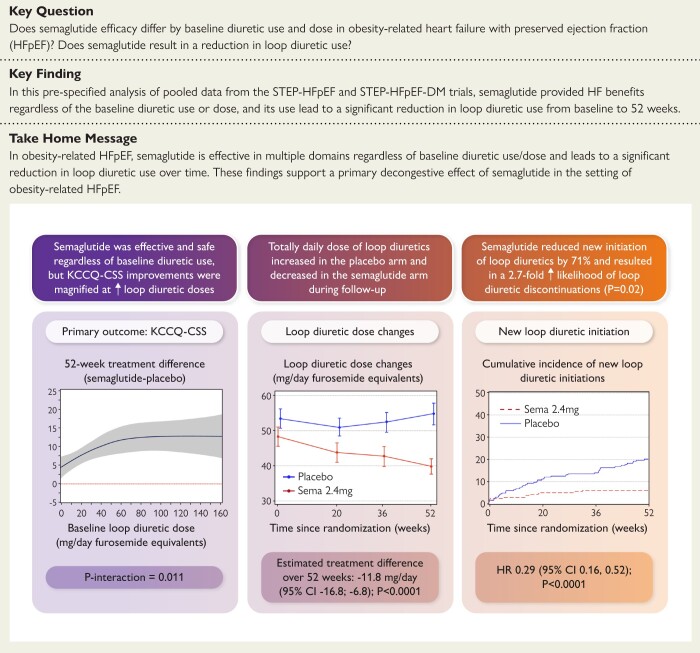
Semaglutide was effective and safe regardless of baseline loop diuretic dose, though health status benefits were magnified at higher loop diuretic doses. Total daily loop diuretic dose decreased in the semaglutide-treated patients, and new loop diuretic initiation was reduced by 71% in the semaglutide-treated patients compared to placebo. CI, confidence interval; HR, hazard ratio; KCCQ-CSS, Kansas City Cardiomyopathy Questionnaire clinical summary score; Sema, semaglutide.

**See the editorial comment for this article ‘STEPping down diuretic therapy with semaglutide in obesity-related heart failure with preserved ejection fraction: decongestion or disease modification?’, by J.W. Ostrominski *et al.*, https://doi.org/10.1093/eurheartj/ehae410.**

## Introduction

Patients with heart failure (HF) and preserved ejection fraction (HFpEF) frequently receive loop diuretics, which are first-line agents for decongestion but can cause electrolyte abnormalities, worsening kidney function, and hypotension.^1^ Furthermore, outpatient escalation in loop diuretic dose is associated with adverse outcomes and is increasingly viewed as a proxy for HF hospitalizations.^2–9^ In HFpEF, higher body mass index is associated with greater use and doses of loop diuretics;^10,11^ and in patients with obesity-related HFpEF, loop diuretics appear to be less effective for decongestion and have an exaggerated unfavourable impact on kidney function, as compared with those that have HFpEF but no obesity.^12^ In the STEP-HFpEF trial programme (which included STEP-HFpEF and STEP-HFpEF-DM trials), the glucagon-like peptide-1 receptor agonist (GLP1-RA) semaglutide, at a weight management dose of 2.4 mg once weekly, improved HF-related symptoms and physical limitations [as measured by the Kansas City Cardiomyopathy Questionnaire clinical summary score (KCCQ-CSS)], reduced body weight, improved exercise function [6 min walk distance (6MWD)], and reduced markers of inflammation (C-reactive protein) and myocardial end-diastolic wall stress [N-terminal pro-B-type natriuretic peptide (NT-proBNP)] in obesity-related HFpEF compared with placebo.^13–15^ Use of semaglutide also led to fewer adjudicated HF events (pooled hazard ratio (HR) 0.27, 95% confidence interval [CI] 0.12–0.56; *P* = .0004).^15^

Whether the effects of semaglutide vary according to baseline diuretic use and dose, and whether semaglutide (as compared with placebo) has an effect on loop diuretic use and dose over time are important, clinically relevant questions. We therefore examined whether semaglutide efficacy varies according to baseline diuretic use, and whether semaglutide results in changes in loop diuretic use and dose over time in a pre-specified analysis of pooled data from the STEP-HFpEF and STEP-HFpEF-DM trials.

## Methods

### Trial programme design

We conducted a pre-specified analysis of the randomized, international, multicentre, double-blind, placebo-controlled STEP-HFpEF programme.^13–15^ The programme comprised two trials: STEP-HFpEF, which was conducted in patients with obesity-related HFpEF [body mass index (BMI) ≥ 30 kg/m^2^, left ventricular ejection fraction (LVEF) ≥ 45%] without type 2 diabetes (NCT04788511);^13^ and STEP-HFpEF-DM in patients with obesity-related HFpEF and type 2 diabetes (NCT04916470).^14^ The design and primary results of the individual trials, and the overall programme, have been published previously.^13,14,16^ The two trials were conducted from 2021 to 2023 at 129 sites across 18 countries in Asia, Europe, and North and South America. Institutional Review Board ethics approval was obtained at each study site, and all patients provided written, informed consent to participate in the trial. The steering committee, which included both academic members and representatives from the sponsor (Novo Nordisk), designed both trials and was responsible for the academic publications. A global expert panel provided academic, medical, and operational input in each country. The sponsor of the trial programme was Novo Nordisk.

### Trial participants

Participants were eligible if they had symptomatic HF, LVEF ≥ 45%, BMI ≥ 30 kg/m^2^, New York Heart Association (NYHA) functional class II–IV, KCCQ-CSS < 90 points, and at least one of the following: (i) elevated filling pressures (based on right heart catheterization or pulmonary artery pressure sensor technology); (ii) elevated natriuretic peptide levels (with thresholds stratified based on BMI) plus echocardiographic abnormalities; or (iii) HF hospitalization in the previous 12 months plus a requirement for ongoing diuretic treatment and/or echocardiographic abnormalities.

Key exclusion criteria were prior or planned bariatric surgery, self-reported change in body weight > 5 kg within 90 days before randomization, or a systolic blood pressure of >160 mmHg at screening. In STEP-HFpEF-DM, patients with uncontrolled diabetic retinopathy or maculopathy were also excluded.

### Randomization and trial procedures

Eligible participants were randomized 1:1 to receive a once-weekly target dose of semaglutide 2.4 mg subcutaneously or matching placebo on top of standard of care for 52 weeks. Randomization was stratified by BMI < 35 kg/m^2^ vs. ≥35 kg/m^2^. Among patients with type 2 diabetes enrolled in the STEP-HFpEF-DM trial, semaglutide or placebo was added to background glucose-lowering medications, which could comprise any drug other than GLP1-RAs. Modification of glucose-lowering treatment was at the discretion of the investigator. Specific guidance regarding the adjustment of sulfonylurea and insulin doses was provided to mitigate the risk of hypoglycaemia. The management of diuretic therapy was left to the discretion of the investigators and treating physicians; no specific instructions regarding the use and dose of diuretics were provided. Detailed ascertainment of diuretic use and types, and doses of loop diuretics, was conducted at baseline, 20-, 36-, and 52-week visits in both trials. Loop diuretics, thiazide diuretics, and mineralocorticoid receptor antagonists (MRAs) were considered diuretics; and sodium–glucose cotransporter 2 (SGLT2) inhibitors were not considered diuretics for the current analysis. All non-furosemide loop diuretic doses were converted to mg/day furosemide equivalents based on published equivalent dose conversions, as listed in Supplementary data online, *Table S1*.^17^ Participants not taking loop diuretics at baseline or at subsequent follow-up visits were coded using a dose of 0 mg/day furosemide equivalents.

### Outcomes

The primary aims of the current analysis were to investigate the effects of semaglutide 2.4 mg once weekly, compared with placebo, on (i) efficacy and safety outcomes across baseline diuretic use groups (no diuretics, non-loop diuretics only, and loop diuretics [<40, 40, and >40 mg/day furosemide equivalents]); and (ii) changes in loop diuretic use and dose over 52 weeks.

The dual primary endpoints of the STEP-HFpEF programme were: (i) change in KCCQ-CSS from baseline to 52 weeks; and (ii) per cent change in body weight from baseline to 52 weeks. The confirmatory secondary endpoints were change in 6MWD from baseline to 52 weeks; a hierarchical composite endpoint [comprised of all-cause death (from baseline to 57 weeks), HF events (from baseline to 57 weeks), differences in several thresholds (≥5, ≥ 10, ≥ 15 points) of change in KCCQ-CSS from baseline to 52 weeks, and difference in 6MWD change (≥30 m) from baseline to 52 weeks]; and change in high-sensitivity C-reactive protein from baseline to 52 weeks. Heart failure events were adjudicated by a blinded Clinical Events Committee as previously described.^13^ We also examined the additional supportive secondary endpoints, including change in systolic blood pressure, waist circumference, and KCCQ overall summary score; and exploratory endpoints of change in NT-proBNP levels, and change in the additional KCCQ domains [total symptom score, physical limitations score (PLS), social limitations score, and quality of life score] from baseline to 52 weeks. Safety endpoints included in the current analysis were serious adverse events (SAEs), which included SAEs leading to permanent treatment discontinuation, cardiac and gastrointestinal SAEs; and gastrointestinal adverse events leading to discontinuation of study drug.

### Statistical analysis

Baseline characteristics were examined according to the five aforementioned baseline diuretic use/dose groups. The Jonckheere–Terpstra trend test (for continuous variables), the Cochran–Armitage trend test (for categorical variables), and Cochran–Mantel–Haenszel test (for multinomial variables) were used to evaluate differences among the five groups.

All efficacy analyses (including change in loop diuretic use and dose) were done using the full analysis set (all randomized participants according to the intention-to-treat principle, while in trial, regardless of treatment discontinuation). For change in KCCQ scores and 6MWD, missing observations at Week 52 caused by cardiovascular death or previous HF events were single imputed to the lowest observed value across both treatment arms and visits. Missing values due to other reasons were imputed using multiple imputation from participants with non-missing values in the same randomized treatment arm. For other endpoints, missing observations at Week 52 were multiple imputed irrespective of death or prior HF events using the same imputation method. Analyses were performed using analysis of covariance (ANCOVA) models for continuous endpoints, with change in the corresponding endpoint at Week 52 as the dependent variable, with adjustment for the baseline value of the relevant continuous outcome variable, and treatment, trial, and BMI stratification as fixed factors using 1000 imputations. To determine whether efficacy endpoints were consistent across the diuretic groups, we included a diuretic group × treatment interaction term in all models. Estimates were then combined using Rubin’s rule. Interaction *P*-values were derived from an *F*-test of equality between the treatment differences across diuretic groups. Furthermore, trend *P*-values for difference in semaglutide vs. placebo treatment across the diuretic medication groups were also derived for the various endpoints. In sensitivity analyses, these analyses were repeated using the comparisons of (i) no diuretic vs. any diuretic use at baseline; and (ii) no loop diuretic vs. loop diuretic (any dose) at baseline.

To further explore the relationship between baseline loop diuretic dose and key efficacy endpoints (changes in KCCQ-CSS, body weight, 6MWD, high-sensitivity C-reactive protein, NT-proBNP), interaction *P*-values between the loop diuretic dose as a continuous variable (modelled as a spline) and randomized treatment at Week 52 were derived to assess potential heterogeneity of treatment effects (semaglutide vs. placebo) across the range of loop diuretic doses.

Analyses of the hierarchical composite endpoint (win ratio^18^) were performed stratified by diuretic use groups, based on direct comparisons of each participant randomized to semaglutide vs. each participant randomized to placebo. For each of the participant pairs, a ‘treatment winner’ based on similar observation time was declared based on the endpoint hierarchy. The win ratio (i.e. the proportion of winners randomized to semaglutide divided by the winners randomized to placebo) was estimated independently within each diuretic group (using 1000 imputations as described above). A win ratio of 1 indicates no difference between treatment groups; a win ratio > 1 favours the active treatment; and a win ratio < 1 favours the placebo. The test for equality of the diuretic groups for the win ratio was performed using a Cochran’s *Q* test.

Safety endpoints across the diuretic groups were analysed using the safety analysis set (all randomized participants exposed to at least one dose of randomized treatment) and either on-treatment or in-trial data sets, depending on the type of safety event.

The difference between treatment groups for change in loop diuretic dose from baseline to Week 52 was calculated using an ANCOVA model, with trial, treatment, and BMI as fixed factors, and adjusted for baseline loop diuretic dose. Logistic regression was used to determine the odds ratio (OR) and 95% CI for baseline to 52-week increase or decrease in loop diuretic dose, with trial, treatment, and BMI as fixed factors, and adjusted for baseline loop diuretic dose. Supportive analyses were performed for baseline to 20-week, and baseline to 36-week increase or decrease in loop diuretic dose, using the same methodology. Besides changes in loop diuretic doses between baseline and 52 weeks, a new start of loop diuretic was considered a dose increase, and discontinuation of loop diuretics was considered a dose decrease, and patients who died, withdrew from the study or were lost to follow-up, were excluded from these analyses. Baseline characteristics were compared across the three loop diuretic change groups (dose decrease, no change, dose increase) using the same analytic techniques as those described above for the comparison of baseline characteristics across diuretic groups. Logistic regression was then used to determine baseline characteristics associated with diuretic dose escalation.

No adjustment for multiple testing was performed. A two-sided *P*-value of <.05 was considered significant in all analyses except interaction testing, in which an interaction *P-*value < .10 was considered significant. Results are presented as estimated changes from baseline to Week 52 for continuous endpoints or a win ratio (for the hierarchical composite endpoint), with a 95% CI and a two-sided *P*-value. NT-proBNP and high-sensitivity C-reactive protein were log-transformed, and hence, treatment ratios at Week 52 are reported. Analyses were conducted by the independent statistical group at Saint Luke’s Mid America Heart Institute in collaboration with Novo Nordisk, using SAS vs. 9.4 (SAS Institute, Cary, NC, USA). All analyses were performed on anonymized data.

## Results

### Baseline characteristics

A total of 1145 patients were randomized across the STEP-HFpEF programme (*n* = 529 in STEP-HFpEF and *n* = 616 in STEP-HFpEF-DM). At baseline, 220 (19.2%) of the participants were taking no diuretics, 223 (19.5%) were taking non-loop diuretic(s) only, and the remaining 702 (61.3%) were taking loop diuretics. Of the total 1145 trial participants, 219 (19.1%), 309 (30.0%), and 174 (15.2%) were taking a loop diuretic dose of <40, 40, and >40 mg/day furosemide equivalents, respectively. Supplementary data online, *Figure S1* displays the distribution of loop diuretic doses at baseline. *Table 1* displays the baseline characteristics, stratified by baseline diuretic use/dose. Across diuretic groups, there was a stepwise increase in proportion of White participants, NYHA functional class III symptoms, hypertension, and atrial fibrillation. Body mass index, waist circumference, high-sensitivity C-reactive protein, and NT-proBNP values also increased in a stepwise fashion from no diuretics to the highest loop diuretic dose category. Greater loop diuretic use/dose was associated with lower LVEF, KCCQ-CSS, and 6MWD. There were no differences in SGLT2 inhibitor use and angiotensin receptor–neprilysin inhibitor use across diuretic groups. MRA use was highest in the highest loop diuretic dose group. Insulin use increased, and dipeptidyl peptidase-4 inhibitors decreased, in a stepwise fashion from no diuretics to the highest dose loop diuretic group.

**Table 1:** Baseline characteristics of patients from the pooled STEP-HFpEF and STEP-HFpEF-DM trials, stratified by baseline diuretic use

Baseline characteristics^a^	Diuretic group					*P*-value
	No diuretic (*n* = 220)	Non-loop diuretic only (*n* = 223)	Loop diuretic, dose < 40 mg/day (*n* = 219)	Loop diuretic, dose = 40 mg/day (*n* = 309)	Loop diuretic, dose > 40 mg/day (*n* = 174)	
Female sex, *n* (%)	114 (51.8)	108 (48.4)	120 (54.8)	157 (50.8)	71 (40.8)	.14
Age, years, *n* (%)						.061
<65	88 (40.0)	69 (30.9)	65 (29.7)	97 (31.4)	49 (28.2)	
65–79	116 (52.7)	140 (62.8)	131 (59.8)	174 (56.3)	105 (60.3)	
≥80	16 (7.3)	14 (6.3)	23 (10.5)	38 (12.3)	20 (11.5)	
Race,^b^*n* (%)						<.001
Asian	38 (17.3)	14 (6.3)	15 (6.8)	8 (2.6)	1 (0.6)	
Black or African American	6 (2.7)	12 (5.4)	4 (1.8)	14 (4.5)	3 (1.7)	
Other	0 (0.0)	1 (0.4)	3 (1.4)	0 (0.0)	0 (0.0)	
White	176 (80.0)	196 (87.9)	197 (90.0)	287 (92.9)	170 (97.7)	
Body weight, kg	99.2 (90.4, 112.7)	101.3 (89.5, 115.5)	100.5 (87.4, 116.0)	106.2 (96.0, 125.1)	110.4 (97.2, 122.9)	<.001
BMI, kg/m^2^	37.2 (34.1, 41.3)	37.3 (34.3, 40.6)	36.9 (33.6, 41.3)	39.3 (35.4, 43.7)	39.2 (35.3, 44.9)	<.001
Waist circumference, cm	117.0 (108.3, 126.0)	118.4 (111.0, 127.1)	118.0 (108.5, 126.0)	122.0 (114.0, 132.0)	124.8 (114.7, 134.6)	<.001
Systolic BP, mmHg	133.0 (123.0, 145.0)	137.0 (127.0, 146.0)	134.0 (122.0, 141.0)	133.0 (124.0, 144.0)	130.0 (120.0, 144.0)	.010
NYHA class, *n* (%)						<.001
II	171 (77.7)	170 (76.2)	149 (68.0)	197 (63.8)	98 (56.3)	
III	49 (22.3)	53 (23.8)	70 (32.0)	110 (35.6)	76 (43.7)	
IV	0 (0.0)	0 (0.0)	0 (0.0)	2 (0.6)	0 (0.0)	
LVEF, %	60.0 (55.0, 61.0)	57.0 (50.0, 60.0)	55.0 (50.0, 60.0)	56.0 (51.0, 60.0)	55.0 (50.0, 61.0)	<.001
KCCQ-CSS, score	65.1 (50.5, 77.1)	65.4 (49.5, 77.1)	57.3 (44.3, 69.8)	55.7 (39.6, 68.2)	50.0 (35.4, 66.7)	<.001
6MWD, metres	336.3 (249.3, 399.8)	338.8 (261.5, 382.0)	288.9 (220.0, 362.0)	265.0 (202.0, 340.0)	260.6 (192.0, 340.0)	<.001
CRP, mg/L	3.4 (1.6, 8.0)	2.7 (1.6, 5.9)	3.2 (1.7, 6.5)	4.7 (2.2, 9.7)	4.2 (2.1, 9.5)	<.001
NT-proBNP, pg/mL	338.3 (207.2, 849.1)	392.4 (208.9, 807.6)	463.4 (258.8, 981.9)	500.7 (244.8, 1091.0)	785.7 (303.6, 1407.2)	<.001
Comorbidities at screening, *n* (%)						
Hypertension	153 (69.5)	201 (90.1)	192 (87.7)	262 (84.8)	151 (86.8)	<.001
Atrial fibrillation	74 (33.6)	89 (39.9)	101 (46.1)	155 (50.2)	99 (56.9)	<.001
Obstructive sleep apnoea	17 (7.7)	26 (11.7)	19 (8.7)	32 (10.4)	25 (14.4)	.11
Coronary artery disease	98 (44.5)	89 (39.9)	95 (43.4)	99 (32.0)	72 (41.4)	.077
Diabetes	118 (53.6)	125 (56.1)	124 (56.6)	158 (51.1)	91 (52.3)	.44
Concomitant medications, *n* (%)						
Beta blockers	164 (74.5)	187 (83.9)	182 (83.1)	251 (81.2)	144 (82.8)	.11
SGLT2 inhibitors	35 (15.9)	44 (19.7)	52 (23.7)	52 (16.8)	38 (21.8)	.40
MRAs	0 (0.0)	89 (39.9)	85 (38.8)	119 (38.5)	91 (52.3)	<.001
Thiazide diuretics	0 (0.0)	102 (45.7)	23 (10.5)	31 (10)	19 (10.9)	.052
ACEi/ARB (ARNi)	149 (67.7)	194 (87.0)	180 (82.2)	246 (79.6)	130 (74.7)	.32
ARNi	4 (1.8)	13 (5.8)	18 (8.2)	13 (4.2)	10 (5.7)	.22
Insulin and analogues	19 (8.6)	20 (9.0)	24 (11.0)	37 (12.0)	28 (16.1)	.013
Sulfonylureas	19 (8.6)	26 (11.7)	29 (13.2)	19 (6.1)	15 (8.6)	.35
DPP-4 inhibitors	27 (12.3)	19 (8.5)	16 (7.3)	19 (6.1)	11 (6.3)	.011

Percentages may not equal 100% due to rounding. Data are median (Q1, Q3) unless otherwise stated and are from the full analysis set. *P*-values for continuous variables computed using the Jonckheere–Terpstra trend test for continuous variables, the Cochran–Armitage trend test for binary variables, and the Cochran–Mantel–Haenszel test for multinomial variables.

6MWD, 6 min walking distance; ACEi, angiotensin-converting enzyme inhibitor; ARB, angiotensin receptor blocker; ARNi, angiotensin receptor–neprilysin inhibitor; BMI, body mass index; CRP, high-sensitivity C-reactive protein; DM, diabetes mellitus; DPP-4, dipeptidyl peptidase-4; KCCQ-CSS, Kansas City Cardiomyopathy Questionnaire clinical summary score; LVEF, left ventricular ejection fraction; MRA, mineralocorticoid receptor antagonist; NT-proBNP, N-terminal pro-B-type natriuretic peptide; NYHA, New York Heart Association; Q, quartile; SGLT2, sodium–glucose cotransporter 2.

^a^A total of 1146 participants were randomized; however, one participant was randomized in error such that the full analysis set comprises 1145 participants.

^b^Race was reported by the investigator.

### Effects of semaglutide vs. placebo on efficacy and safety endpoints by baseline diuretic use

The effects of semaglutide on the dual primary, confirmatory secondary, supportive secondary, and exploratory endpoints across the diuretic use subgroups are presented in *Table 2*. The benefits of semaglutide were consistent across the health status, body weight, exercise function, and biomarker endpoints across the diuretic use categories [interaction *P* > .10 for all endpoints except for KCCQ-PLS (interaction *P* = .092); *Table 2*]. However, progressively larger increases (improvements) in the KCCQ-CSS (and PLS) domains occurred with semaglutide vs. placebo from the no diuretic subgroup to the highest dose loop diuretic subgroup (*Table 2*). These results were verified in sensitivity analyses comparing (i) no diuretic vs. any diuretic groups (see Supplementary data online, *Table S2*) and (ii) no loop diuretic vs. loop diuretic (any dose) groups (see Supplementary data online, *Table S3*), which demonstrate that KCCQ-CSS improvements were larger in patients on any diuretic (compared to no diuretic), and any dose of loop diuretic (compared to no loop diuretic), in particular (adjusted mean KCCQ-CSS change for semaglutide vs. placebo: +9.3 points [6.5, 12.1] in participants on loop diuretics at baseline vs. +4.7 points [1.3, 8.2] in participants not on loop diuretics at baseline; *P* for interaction = .042). Win ratios were similar across diuretic use groups (no diuretics [1.17 (95% CI 0.85–1.63)]; non-loop diuretics only [1.64 (95% CI 1.17–2.30)]; <40 mg/day loop diuretic [1.73 (95% CI 1.19–2.52)]; 40 mg/day loop diuretic [1.79 (95% CI 1.34–2.40)]; and >40 mg loop diuretic [2.06 (95% CI 1.39–3.06)]), with no treatment heterogeneity (interaction *P* = .24), indicating that semaglutide had consistent efficacy on the hierarchical composite endpoint across diuretic groups.

**Table 2:** Effect of semaglutide vs. placebo on efficacy endpoints, stratified by baseline diuretic use pooled across the STEP-HFpEF and STEP-HFpEF-DM trials

Endpoint	Parameter	No diuretics		Non-loop diuretic only		Loop diuretic, dose < 40 mg		Loop diuretic, dose = 40 mg		Loop diuretic, dose > 40 mg		Interaction *P*-value	*P*-value for trend
		Sema (*n* = 120)	Placebo (*n* = 100)	Sema (*n* = 109)	Placebo (*n* = 114)	Sema (*n* = 110)	Placebo (*n* = 109)	Sema (*n* = 152)	Placebo (*n* = 157)	Sema (*n* = 82)	Placebo (*n* = 92)		
Dual primary endpoints													
KCCQ-CSS, points	52-Week change	14.4 (11.0, 17.7)	11.2 (7.5, 14.9)	15.8 (12.3, 19.2)	9.5 (6.1, 12.9)	15.4 (11.9, 19.0)	7.5 (4.0, 11.0)	15.0 (12.0, 18.0)	6.2 (3.2, 9.1)	14.6 (10.5, 18.7)	3.0 (−0.8, 6.8)	.22	.02
	Adjusted mean difference	3.2 (−1.8, 8.2)		6.2 (1.4, 11.1)		7.9 (2.9, 12.9)		8.9 (4.7, 13.0)		11.6 (6.0, 17.2)			
Body weight, %	52-Week change, %	−11.4 (−12.7, −10.1)	−3.0 (−4.6, −1.5)	−11.8 (−13.2, −10.5)	−3.0 (−4.4, −1.7)	−10.6 (−12.0, −9.2)	−3.0 (−4.4, −1.6)	−12.0 (−13.2, −10.8)	−2.6 (−3.7, −1.4)	−10.7 (−12.4, −9.1)	−3.8 (−5.3, −2.3)	.39	.50
	Adjusted mean difference, %	−8.3 (−10.3, −6.3)		−8.8 (−10.7, −6.9)		−7.6 (−9.5, −5.6)		−9.4 (−11.1, −7.8)		−6.9 (−9.1, −4.7)			
Confirmatory secondary endpoints													
6MWD, m	52-Week change, m	18.4 (6.6, 30.1)	0.4 (−12.9, 13.6)	22.3 (10.0, 34.7)	1.5 (−10.5, 13.6)	10.9 (−1.8, 23.6)	4.9 (−7.6, 17.4)	18.8 (8.4, 29.3)	1.5 (−9.1, 12.1)	11.2 (−3.6, 26.0)	−12.8 (−26.3, 0.7)	.70	.77
	Adjusted mean difference, m	18.0 (0.4, 35.7)		20.8 (3.6, 37.9)		6.0 (−11.8, 23.8)		17.3 (2.5, 32.1)		24.0 (3.9, 44.0)			
Hierarchical composite endpoint	Win ratio	1.17 (0.85–1.63)		1.64 (1.17–2.30)		1.73 (1.19–2.52)		1.79 (1.34–2.40)		2.06 (1.39–3.06)		.24	
CRP, mg/L	52-Week ratio	0.53 (0.44, 0.63)	0.88 (0.71, 1.08)	0.62 (0.51, 0.75)	0.84 (0.70, 1.01)	0.53 (0.43, 0.65)	0.79 (0.65, 0.95)	0.58 (0.49, 0.69)	0.95 (0.81, 1.11)	0.62 (0.49, 0.80)	1.06 (0.86, 1.31)	.76	.60
	Treatment ratio	0.60 (0.46, 0.79)		0.74 (0.57, 0.96)		0.68 (0.51, 0.90)		0.61 (0.48, 0.77)		0.59 (0.43, 0.81)			
Supportive secondary endpoints													
Systolic BP, mmHg	52-Week change, mmHg	−1.7 (−4.6, 1.2)	−0.3 (−3.8, 3.2)	−4.1 (−7.1, −1.1)	−1.8 (−4.9, 1.2)	−6.0 (−9.1, −2.8)	−1.8 (−4.9, 1.4)	−5.0 (−7.6, −2.4)	−1.6 (−4.2, 1.1)	−7.1 (−10.7, −3.4)	−3.4 (−7.0, 0.1)	.91	.45
	Adjusted mean diff., mmHg	−1.4 (−5.9, 3.2)		−2.3 (−6.6, 2.1)		−4.2 (−8.7, 0.3)		−3.4 (−7.1, 0.3)		−3.6 (−8.7, 1.5)			
Waist circumference, cm	52-Week change, cm	−10.7 (−12.1, −9.2)	−3.3 (−5.1, −1.6)	−10.4 (−12.0, −8.8)	−2.8 (−4.4, −1.3)	−9.6 (−11.2, −8.0)	−2.7 (−4.3, −1.1)	−10.6 (−12.0, −9.2)	−2.4 (−3.7, −1.0)	−9.7 (−11.6, −7.7)	−2.0 (−3.8, −0.2)	.92	.70
	Adjusted mean difference, cm	−7.3 (−9.6, −5.0)		−7.6 (−9.8, −5.4)		−6.9 (−9.2, −4.6)		−8.3 (−10.2, −6.3)		−7.7 (−10.3, −5.1)			
KCCQ-OSS, points	52-Week change	14.7 (11.3, 18.0)	10.7 (7.1, 14.4)	15.6 (12.1, 19.0)	9.5 (6.1, 12.9)	15.5 (11.9, 19.1)	7.2 (3.7, 10.6)	14.6 (11.6, 17.6)	7.0 (4.1, 9.9)	14.4 (10.3, 18.6)	2.8 (−1.0, 6.6)	.33	.04
	Adjusted mean difference	3.9 (−1.0, 8.9)		6.1 (1.2, 11.0)		8.3 (3.3, 13.3)		7.6 (3.5, 11.8)		11.7 (6.1, 17.3)			
Exploratory endpoints													
NT-proBNP, pg/mL	52-Week ratio	0.70 (0.60, 0.81)	0.93 (0.77, 1.12)	0.73 (0.62, 0.86)	0.98 (0.83, 1.16)	0.78 (0.66, 0.92)	0.83 (0.70, 0.98)	0.83 (0.72, 0.95)	0.95 (0.83, 1.10)	0.89 (0.73, 1.07)	1.11 (0.92, 1.33)	.54	.45
	Treatment ratio	0.75 (0.59, 0.95)		0.74 (0.59, 0.93)		0.95 (0.75, 1.20)		0.87 (0.71, 1.06)		0.80 (0.62, 1.04)			
KCCQ-TSS, points	52-Week change	15.8 (12.3, 19.3)	10.3 (6.4, 14.3)	16.2 (12.5, 19.9)	8.3 (4.7, 12.0)	15.9 (12.2, 19.7)	9.0 (5.3, 12.7)	16.2 (13.0, 19.3)	7.0 (3.9, 10.1)	15.0 (10.7, 19.3)	2.7 (−1.4, 6.8)	.51	.09
	Adjusted mean difference	5.5 (0.2, 10.7)		7.8 (2.7, 13.0)		6.9 (1.6, 12.2)		9.1 (4.7, 13.5)		12.3 (6.4, 18.2)			
KCCQ-PLS, points	52-Week change	13.5 (9.8, 17.2)	11.9 (7.7, 16.1)	15.5 (11.7, 19.3)	11.4 (7.6, 15.2)	15.2 (11.2, 19.1)	5.9 (2.0, 9.8)	14.1 (10.8, 17.4)	4.5 (1.2, 7.8)	13.1 (8.5, 17.6)	2.5 (−1.8, 6.7)	.092	.01
	Adjusted mean difference	1.6 (−4.0, 7.1)		4.1 (−1.3, 9.5)		9.3 (3.7, 14.8)		9.6 (4.9, 14.2)		10.6 (4.4, 16.8)			
KCCQ-SLS, points	52-Week change	15.3 (11.1, 19.4)	10.6 (5.9, 15.2)	14.9 (10.5, 19.3)	10.8 (6.6, 15.1)	15.7 (11.3, 20.1)	6.4 (2.1, 10.8)	11.9 (8.2, 15.7)	6.8 (3.1, 10.5)	11.7 (6.5, 16.9)	−0.1 (−4.9, 4.7)	.39	.14
	Adjusted mean difference	4.7 (−1.5, 10.9)		4.1 (−2.0, 10.2)		9.2 (3.0, 15.4)		5.2 (−0.1, 10.4)		11.7 (4.7, 18.8)			
KCCQ-QLS, points	52-Week change	15.6 (11.8, 19.4)	10.9 (6.7, 15.2)	15.8 (11.9, 19.8)	9.5 (5.6, 13.5)	15.3 (11.2, 19.3)	7.3 (3.2, 11.3)	15.6 (12.2, 19.0)	8.4 (5.0, 11.8)	15.0 (10.3, 19.6)	4.3 (−0.0, 8.7)	.72	.17
	Adjusted mean difference	4.7 (−1.1, 10.4)		6.3 (0.7, 11.9)		8.0 (2.3, 13.7)		7.2 (2.4, 12.0)		10.6 (4.2, 17.0)			

**Figure 1:**
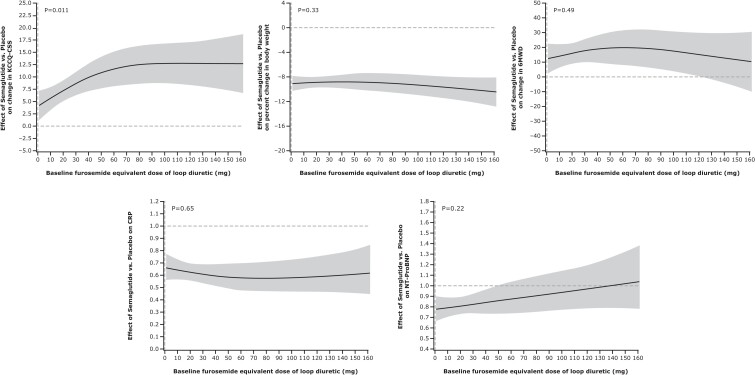
Restricted cubic spline curves of key efficacy outcomes over the range of baseline daily loop diuretic dose pooled across the STEP-HFpEF and STEP-HFpEF-DM trials. Loop diuretic dose expressed in mg furosemide equivalents per day. 6MWD, 6 min walk distance; CRP, C-reactive protein; KCCQ-CSS, Kansas City Cardiomyopathy Questionnaire clinical summary score; NT-proBNP, N-terminal pro-B-type natriuretic peptide; w, week

*Figure 1* displays the restrictive cubic spline curves of key efficacy outcomes over the range of baseline loop diuretic dose (total daily mg furosemide equivalents). When examined as a continuous variable, KCCQ-CSS improvement was greater in the semaglutide vs. placebo groups with increasing doses of loop diuretics (*P* = .026). No significant interactions were observed for changes in body weight, 6MWD, C-reactive protein, or NT-proBNP.

There were fewer SAEs, and cardiac SAEs, in patients treated with semaglutide vs. placebo across all diuretic groups (*Table 3*). Gastrointestinal SAEs occurred at similar rates in the semaglutide and placebo groups across diuretic groups (*Table 3*).

**Table 3:** Serious adverse events stratified by baseline diuretic use, pooled across the STEP-HFpEF and STEP-HFpEF-DM trials

Adverse event, *n* (%)	Diuretic group									
	No diuretic		Non-loop diuretic		Loop diuretic < 40 mg/day		Loop diuretic = 40 mg/day		Loop diuretic > 40 mg/day	
	Semaglutide *n* = 120	Placebo *n* = 100	Semaglutide *n* = 109	Placebo *n* = 114	Semaglutide *n* = 110	Placebo *n* = 109	Semaglutide *n* = 152	Placebo *n* = 157	Semaglutide *n* = 82	Placebo *n* = 92
Serious adverse events	13 (10.8)	25 (25.0)	16 (14.7)	24 (21.1)	11 (10.0)	24 (22.0)	25 (16.4)	48 (30.6)	25 (30.5)	38 (41.3)
Serious adverse events leading to permanent treatment discontinuation	1 (0.8)	1 (1.0)	3 (2.8)	4 (3.5)	0 (0.0)	2 (1.8)	3 (2.0)	6 (3.8)	5 (6.1)	4 (4.3)
Cardiac serious adverse events	2 (1.7)	12 (12.0)	5 (4.6)	10 (8.8)	5 (4.5)	9 (8.3)	8 (5.3)	22 (14.0)	6 (7.3)	17 (18.5)
Gastrointestinal serious adverse events	0 (0.0)	1 (1.0)	2 (1.8)	2 (1.8)	2 (1.8)	1 (0.9)	5 (3.3)	5 (3.2)	3 (3.7)	3 (3.3)
COVID-19-related adverse events	14 (11.7)	15 (15.0)	13 (11.9)	12 (10.5)	14 (12.7)	14 (12.8)	19 (12.5)	22 (14.0)	13 (15.9)	17 (18.5)

### Effects of semaglutide vs. placebo on loop diuretic dose

**Figure 2:**
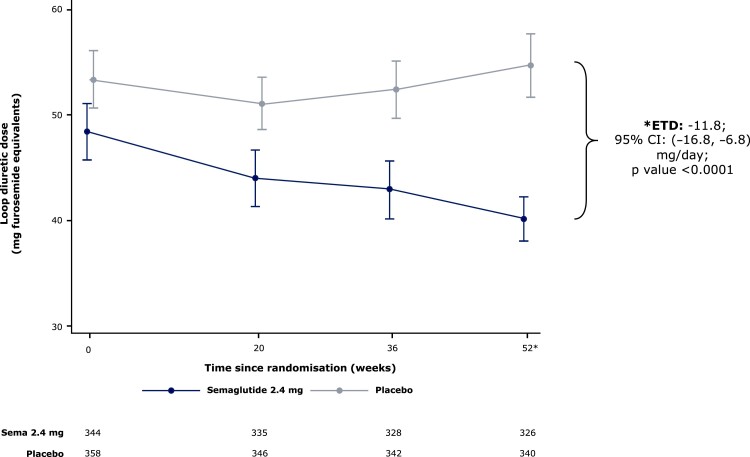
Effect of semaglutide vs. placebo on loop diuretic dose from baseline to 52 weeks, pooled across the STEP-HFpEF and STEP-HFpEF-DM trials. Error bars represent standard deviations. There was no significant difference in baseline loop diuretic dose between the semaglutide and placebo groups (*P* = .19)

**Figure 3:**
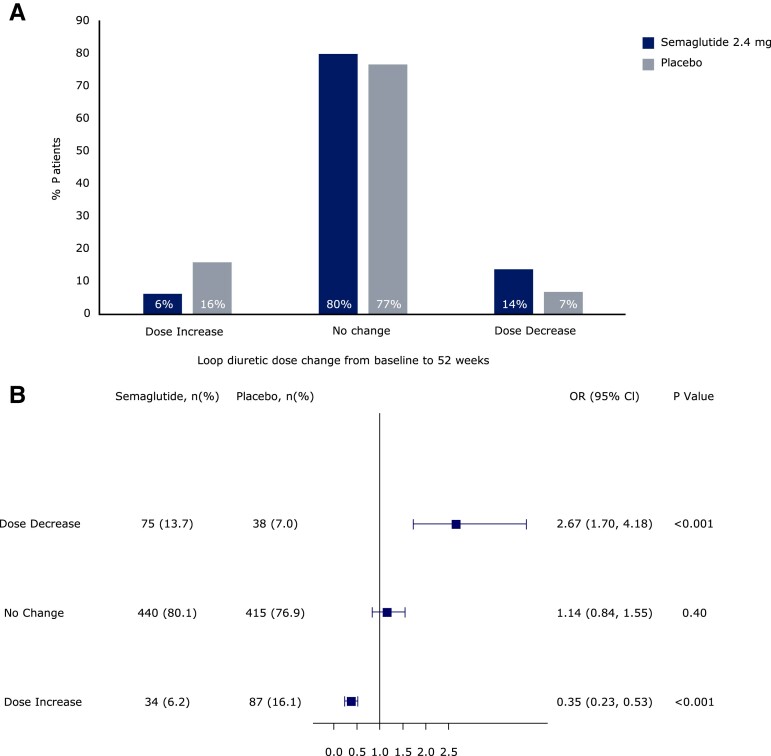
Effect of semaglutide vs. placebo on loop diuretic dose changes from baseline to 52 weeks, pooled across the STEP-HFpEF and STEP-HFpEF-DM trials. Odds of loop diuretic dose changes over 52 weeks in response to treatment with semaglutide 2.4 mg: dose increase (OR 0.34 [95% CI 0.23–0.52]), *P* < .001; dose decrease (OR 2.09 [95% CI 1.39–3.15], *P* < .001)

**Figure 4:**
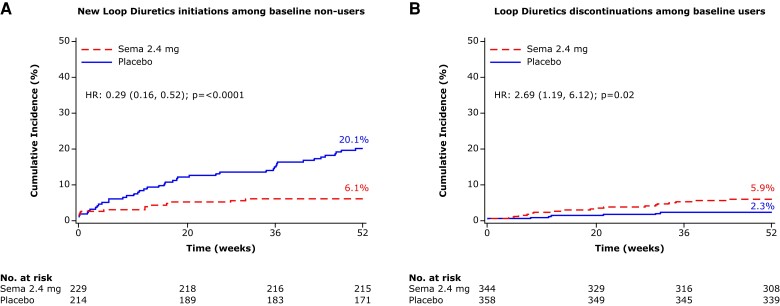
Effects of semaglutide vs. placebo on the time to first loop diuretic start among non-users at baseline (*A*) and time to first loop diuretic stop in users at baseline (*B*), pooled across the STEP-HFpEF and STEP-HFpEF-DM trials

**Figure 5:**
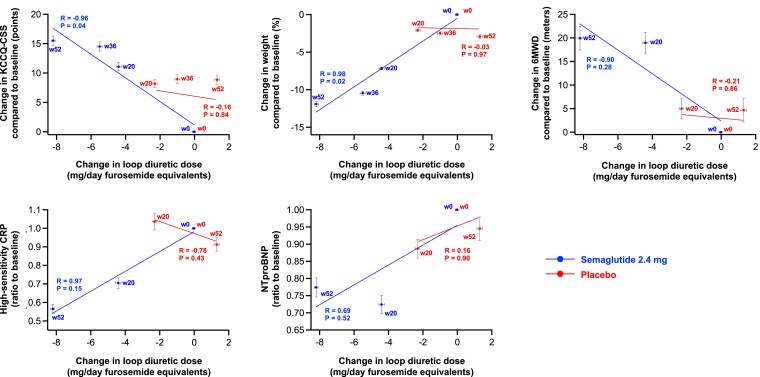
Correlations of group mean changes in loop diuretic dose vs. changes in efficacy endpoints at baseline and follow-up time points, stratified by treatment group, pooled across the STEP-HFpEF and STEP-HFpEF-DM trials. *R*-values represent Pearson correlation coefficients, and error bars represent standard errors. 6MWD, 6 min walk distance; CRP, C-reactive protein; KCCQ-CSS, Kansas City Cardiomyopathy Questionnaire clinical summary score; NT-proBNP, N-terminal pro-B-type natriuretic peptide; w, week

From baseline to 52 weeks, loop diuretic dose decreased by 17% (from mean ± SD 48.4 ± 2.7 to 40.2 ± 2.1 mg/day furosemide equivalents) in the semaglutide group vs. an increase of 2.4% (53.4 ± 2.7 to 54.7 ± 3.1 mg/day) in the placebo group, which resulted in a difference of 11.8 (95% CI 6.8, 16.8) mg/day lower loop diuretic dose in semaglutide vs. placebo groups (*P* < .0001) (*Figure 2*). Compared with placebo, semaglutide-treated patients were also more likely to experience a loop diuretic dose reduction (OR 2.67 [95% CI 1.70, 4.18]) and less likely to experience a dose increase (OR 0.35 [0.23, 0.53]) between baseline and 52 weeks; *P* < .001 for both (*Figure 3*). The results were consistent in the supportive analyses which examined loop diuretic dose reduction and dose increase at 20 weeks, and 36 weeks (see Supplementary data online, *Tables S4A* and *B*, respectively). Semaglutide led to a lower incidence of new starts of loop diuretics in those not on loop diuretics at baseline (HR 0.29 [95% CI 0.16, 0.52]; *P* < .0001) compared with placebo; and higher incidence of stopping loop diuretics in those on loop diuretics at baseline (HR 2.69 [95% CI 1.19, 6.12]; *P* = .02) (*Figure 4*). As shown in *Tables 4* and *5*, participants who required a loop diuretic dose escalation (increase) were more frequently assigned to the placebo group and were more likely to have the following at baseline: NYHA class III/IV symptoms, atrial fibrillation, obstructive sleep apnoea, and treatment with insulin. Higher LVEF, SGLT2 inhibitor use, and renin angiotensin system blockade were all associated with lower likelihood of loop diuretic dose escalation. *Figure 5* displays the correlation between changes in loop diuretic dose over time vs. changes in key efficacy endpoints, stratified by treatment group. There was a linear relationship between reduction in loop diuretic dose and increase (improvement) in KCCQ-CSS and reduction in body weight, and reduction in high-sensitivity C-reactive protein in the semaglutide group.

**Table 4:** Baseline characteristics stratified by loop diuretic dose change over 52 weeks of follow-up

Baseline characteristic	Loop diuretic change group			*P*-value
	Dose decrease (*n* = 113)	No change (*n* = 855)	Dose increase (*n* = 121)	
Female sex, *n* (%)	52 (46.0)	438 (51.2)	55 (45.5)	.90
Age, years, *n* (%)				.07
<65	33 (29.2)	283 (33.1)	33 (27.3)	
65–79	63 (55.8)	500 (58.5)	71 (58.7)	
≥80	17 (15.0)	72 (8.4)	17 (14.0)	
Race,^a^*n* (%)				.10
Asian	5 (4.4)	66 (7.7)	2 (1.7)	
Black or African American	2 (1.8)	30 (3.5)	3 (2.5)	
Other	1 (0.9)	2 (0.2)	1 (0.8)	
White	105 (92.9)	757 (88.5)	115 (95.0)	
Body weight, kg	105.8 (92.0, 121.7)	102.9 (91.0, 117.2)	109.2 (94.0, 124.4)	.69
BMI, kg/m^2^	37.6 (34.1, 42.9)	38.0 (34.5, 42.5)	38.5 (35.5, 43.3)	.23
Waist circumference, cm	122.0 (113.8, 130.3)	119.0 (111.0, 128.5)	123.0 (113.0, 133.0)	.52
Systolic BP, mmHg	130.0 (117.0, 142.5)	134.0 (124.0, 144.0)	135.0 (124.0, 144.0)	.046
NYHA class, *n* (%)				<.001
II	71 (62.8)	613 (71.7)	65 (53.7)	
III	42 (37.2)	240 (28.1)	56 (46.3)	
IV	0 (0.0)	2 (0.2)	0 (0.0)	
LVEF, %	55.0 (50.0, 60.0)	57.0 (51.0, 60.0)	55.0 (50.0, 60.0)	.95
KCCQ-CSS, score	57.3 (41.7, 71.9)	60.4 (44.3, 72.9)	51.0 (33.3, 69.3)	.18
6MWD, metres	260.0 (196.4, 352.0)	300.0 (233.0, 373.0)	265.4 (188.9, 342.0)	.60
CRP, mg/L	3.8 (1.9, 9.4)	3.5 (1.8, 7.6)	3.8 (1.8, 8.1)	.90
NT-proBNP, pg/mL	575.1 (289.2, 1110.4)	426.2 (225.8, 954.8)	749.3 (288.4, 1257.6)	.43
Comorbidities at screening, *n* (%)				
Hypertension	96 (85.0)	711 (83.2)	104 (86.0)	.82
Atrial fibrillation	58 (51.3)	375 (43.9)	67 (55.4)	.48
Obstructive sleep apnoea	16 (14.2)	71 (8.3)	21 (17.4)	.36
Coronary artery disease	40 (35.4)	341 (39.9)	52 (43.0)	.24
Diabetes	40 (35.4)	477 (55.8)	69 (57.0)	.001
Diabetes duration, years	10.4 (4.4, 21.5)	7.9 (4.1, 14.1)	7.1 (3.6, 17.0)	.24
Concomitant medications, *n* (%)				
Beta blockers	14 (12.4)	139 (16.3)	14 (11.6)	.82
SGLT2 inhibitors	95 (84.1)	692 (80.9)	96 (79.3)	.36
MRAs	18 (15.9)	175 (20.5)	19 (15.7)	.93
Thiazide diuretics	49 (43.4)	267 (31.2)	47 (38.8)	.52
ACE inhibitor/ARB (ARNi)	93 (82.3)	678 (79.3)	85 (70.2)	.022
ARNi	8 (7.1)	43 (5.0)	6 (5.0)	.48
Insulin and analogues	8 (7.1)	88 (10.3)	24 (19.8)	.001
Sulfonylureas	9 (8.0)	90 (10.5)	6 (5.0)	.40
DPP-4 inhibitors	8 (7.1)	72 (8.4)	9 (7.4)	.93

Continuous variables are expressed as median (25th–75th percentile). *P*-values for continuous variables computed from Jonckheere–Terpstra trend test; Cochran–Armitage trend test for binary variables; and Cochran–Mantel–Haenszel test for multinomial variables.

^a^Race was reported by the investigator.

**Table 5:** Association of baseline characteristics and treatment group assignment with diuretic dose escalation during 52 weeks of follow-up

Predictor	Odds ratio (95% CI)	*P*-value
Treatment (semaglutide 2.4 mg vs. placebo)	0.36 (0.23–0.55)	<.0001
NYHA class (III/IV vs. II)	1.99 (1.34–2.96)	.0007
LV ejection fraction (per 1%-unit increase)	0.97 (0.95–1.00)	.07
Atrial fibrillation	1.60 (1.07–2.40)	.02
Obstructive sleep apnoea	2.27 (1.31–3.93)	.003
SGLT2 inhibitor use	0.59 (0.34–1.04)	.07
ACE inhibitor/ARB/ARNi use	0.53 (0.34–0.83)	.006
Insulin and analogues	2.91 (1.68–5.05)	.0001

NYHA, New York Heart Association; LV, left ventricular; SGLT2, sodium–glucose cotransporter 2; ACE, angiotensin-converting enzyme; ARB, angiotensin receptor blocker; ARNi, angiotensin receptor–neprilysin inhibitor.

## Discussion

In a pre-specified analysis of the STEP-HFpEF programme, we found that in patients with obesity-related HFpEF, semaglutide 2.4 mg once weekly, compared with placebo, improved HF-related symptoms and physical limitations across diuretic use subgroups, with especially pronounced benefits among patients receiving loop diuretics at baseline. Semaglutide led to consistent beneficial effects on body weight, exercise function, and biomarkers of inflammation and congestion, across the subgroups of background diuretic therapy use and dose. Semaglutide was also consistently well tolerated—with fewer SAEs and cardiac disorders compared with placebo—irrespective of baseline diuretic therapy or dose. Furthermore, during 52 weeks of treatment, compared with placebo, semaglutide treatment led to: (i) a nearly 20% reduction in total daily loop diuretic dose; (ii) more than a two-fold increase in odds of loop diuretic dose reduction; and (iii) 66% lower odds of loop diuretic dose increase. Semaglutide use was also associated with less loop diuretic starts (in those not already on loop diuretics at baseline) and more frequent stopping of loop diuretics (in those on loop diuretics at baseline) compared with placebo (*Structured Graphical Abstract*). In the semaglutide group, reductions in daily loop diuretic dose were also linearly correlated with improvements in health status, reduction in body weight, and reduction in systemic inflammation. These findings demonstrate that semaglutide is effective across the full spectrum of patients with obesity-related HFpEF, from those who do not require loop diuretics to those with significant congestion, requiring high-dose loop diuretic therapy, often with adjunctive MRA and SGLT2 inhibitor use. The results of this study also complement the main findings of the STEP-HFpEF programme, which demonstrated that semaglutide results in disease modification (early and sustained lowering of NT-proBNP; and fewer HF events) by showing that semaglutide leads to clinically relevant and statistically significant reduction in daily loop diuretic dose over time.

Although loop diuretics have been the *de facto* the first-line decongestive treatment for HF across the LVEF spectrum for over 60 years, they can cause electrolyte abnormalities, worsening kidney function, and hypotension.^1^ In obesity-related HFpEF, with the availability of SGLT2 inhibitors, MRAs, and now semaglutide, the need for loop diuretics, particularly at higher doses, may need to be reconsidered and substituted for these agents as first-line therapies. It is notable that in the STEP-HFpEF programme, there were fewer cardiac-related SAEs in the semaglutide group compared with placebo, along with decongestive effects suggested by greater reductions in NT-proBNP, fewer HF events, and lower loop diuretic dose requirements.

The search for alternative decongestive therapies is especially important in patients with obesity-related HFpEF because of the blunted response to loop diuretics in these patients compared to those with HFpEF but no obesity, and the greater frequency of worsening kidney function in patients with obesity-related HFpEF during decongestion.^12^ Reassuringly, the HF benefits and safety of semaglutide were consistent across diuretic use/dose groups; thus, even in patients with minimal congestion, semaglutide is still effective and well tolerated. Nevertheless, it is notable and clinically relevant that for health status (i.e. KCCQ domains), the largest improvements with semaglutide were seen in the most congested patients (i.e. those who required the highest dose of loop diuretics and often were also taking MRAs), who are especially difficult to manage and have few efficacious treatment options. These results also underscore the need for novel non-loop diuretic therapies to effectively decongest patients with obesity-related HFpEF given their propensity for inadequate or poorly tolerated response to loop diuretics.^12^

The discordance between the greater KCCQ benefit in the most congested patients (i.e. those treated with loop diuretics at high doses) despite a similar degree of per cent body weight reduction across the diuretic use/dose groups is of substantial importance. The mean placebo-corrected weight loss was 8.3% vs. 6.9%, whereas the mean placebo-corrected improvement in KCCQ-CSS was 3.2 vs. 11.6 points in the no diuretics and highest dose loop diuretic groups, respectively. These findings, coupled with the reduction in loop diuretic dose over time and the linear relationship between loop diuretic dose reductions and improvements in KCCQ-CSS and C-reactive protein in the semaglutide arm point to potential weight-independent effects of semaglutide on decongestion. Mechanisms underlying these findings remain speculative; possibilities include: (i) selective reduction in epicardial, pericardial, and chest wall adipose tissue (which would reduce pericardial constraint that is present in obesity-related HFpEF, thereby lowering filling pressure;^19^) (ii) weight loss-independent, direct effects of semaglutide on vasorelaxation (via GLP1 receptors on vascular smooth muscle cells;^20,21^) or (iii) beneficial kidney effects of GLP1-RAs, including reduced tubulointerstitial damage, albuminuria, and glomerulosclerosis, with improved podocyte architecture seen in pre-clinical studies,^22^ which may be reasons why semaglutide has led to improved kidney outcomes in several clinical trials,^23^ including the FLOW trial of semaglutide vs. placebo in patients with diabetes and chronic kidney disease, which was stopped early due to overwhelming efficacy.^24^ It is possible that all of these effects may be more pronounced in those patients with the greatest level of congestion at baseline.

Several studies have examined the effects of various HF medications on loop diuretic changes over time in both HFpEF and in HF with reduced ejection fraction.^25–33^ Of the studied HF therapies, MRAs and SGLT2 inhibitors have been the agents with most consistent beneficial effects on the reduction in loop diuretic dose over time; however, these changes have been modest, with lower per cent reduction in doses and lower odds of dose decreases.^25–27,29–31^ For example, in the DELIVER trial, the SGLT2 inhibitor dapagliflozin did not lead to loop diuretic reductions (HR 0.98; 95% CI 0.86–1.13, *P* = .83) during follow-up.^27^

Semaglutide-induced reduction in loop diuretic dose is particularly relevant given the association of outpatient intensification of diuretics with increased risk for subsequent adverse HF events in patients with HF across the LVEF spectrum.^2–8,34^ In addition, multiple studies have demonstrated that higher loop diuretic doses are a proxy for disease severity and are associated with worse outcomes in patients with HF.^35,36^ Given the relationship between high baseline loop diuretic doses and dose intensification with subsequent HF events, it is therefore not surprising that there were fewer HF events observed with semaglutide vs. placebo in the STEP-HFpEF programme (although the programme was not primarily designed to evaluate clinical events).^15^ The 52-week duration of the STEP-HFpEF programme precludes determination of whether the magnitude of reduction in loop diuretic dose is associated with future HF events, and the number of HF events in the overall STEP-HFpEF programme was small. For these reasons, dedicated cardiovascular outcome trials of incretin-based therapies, in patients with obesity-related HFpEF should be undertaken.

### Strengths and limitations

Strengths of this study include the pre-specified nature of the analysis, including harmonization of the endpoints and study procedures across the two trials in the STEP-HFpEF programme, and the detailed recording of diuretic use, types, and doses throughout both trials, which allowed us to examine the effects of semaglutide across groups stratified by baseline diuretic use/dose on a variety of clinically relevant endpoints; and also to determine the effects of semaglutide vs. placebo on loop diuretic doses during the 52-week duration of the trial. Limitations of this study include the analysis of loop diuretic dose changes in isolation, without considering changes in other medications. However, such analyses are challenging given the polypharmacy and hyper-polypharmacy commonly present in HFpEF,^37^ which makes accounting for all medication changes methodologically difficult. We were also unable to compare among the types of loop diuretics given the relatively small sample sizes, although prior studies in HF have found no differences in outcomes in relation to type of loop diuretic.^38^ Nonetheless, our analytic approach was consistent with several other secondary analyses of loop diuretic dose changes in previous HF trials.^25,27–33^ The study is also limited by lack of direct mechanistic insights regarding changes in plasma volume, natriuresis, and cardiac structure/function. The STEP-HFpEF programme does include an echocardiographic substudy, which may shed additional light on the mechanisms behind the benefits of semaglutide in future analyses. Interaction testing may be underpowered and therefore could have missed significant differences in efficacy and safety among diuretic groups. Finally, the STEP-HFpEF programme included a paucity of non-White participants, thereby limiting generalizability.

## Conclusions

In STEP-HFpEF, the first clinical trial programme to examine the role of the GLP1-RA semaglutide in the management of patients with obesity-related HFpEF, the clinical characteristics of patients differed by baseline diuretic use and type, but the majority of beneficial HF-related clinical effects and safety of semaglutide were consistent across diuretic groups, with greater magnitude of improvement in HF-related symptoms and physical limitations in patients taking loop diuretics. Semaglutide treatment led to a clinically meaningful and significant reduction in loop diuretic dose over the 52-week treatment period, which along with reductions in NT-proBNP and fewer HF events, suggests disease-modifying effects in obesity-related HFpEF.

## Supplementary Material

ehae322_Supplementary_Data

## Data Availability

Data will be shared with bona fide researchers submitting a research proposal approved by the independent review board. Access request proposals can be found at novonordisk-trials.com. Data will be made available after research completion, and approval of the product and product use in the European Union and the USA. Individual participant data will be shared in data sets in a de-identified/anonymized format.

## Pre-registered Clinical Trial Number

NCT04788511 and NCT04916470.
