# Supplementary material for: Semaglutide and diuretic use in obesity-related heart failure with preserved ejection fraction: a pooled analysis of the STEP-HFpEF and STEP-HFpEF-DM trials
Source: Eur Heart J. 2024 May 13;45(35):3254–69. doi: 10.1093/eurheartj/ehae322 (PMC11400859; doi:10.1093/eurheartj/ehae322)
Supplement: ehae322_Supplementary_Data [file ehae322_supplementary_data.docx]

**SUPPLEMENTARY MATERIALS**

**Semaglutide and Diuretic Use in Obesity-Related Heart Failure with Preserved Ejection Fraction: A Pooled Analysis of the STEP-HFpEF and STEP-HFpEF-DM trials**

**Sanjiv J. Shah, Kavita Sharma, Barry A. Borlaug, Javed Butler, Melanie Davies,**

**Dalane W Kitzman, Mark C. Petrie, Subodh Verma, Shachi Patel, Khaja M. Chinnakondepalli,**

**Mette N. Einfeldt, Thomas J. Jensen, Søren Rasmussen, Rabea Asleh, Tuvia Ben-Gal,**

**Mikhail N. Kosiborod for the STEP-HFpEF Trial Committees and Investigators**

**Supplemental Table 1. Loop diuretic dose conversion**

Loop diuretic dose conversion formula: 40 mg furosemide = 1 mg bumetanide = 20 mg torsemide = 60 mg azosemide = 50 mg ethacrynic acid

| **Loop diuretic** | **Furosemide-equivalent dose** |
| --- | --- |
| Bumetanide | Dose $\times$ 40 |
| Torsemide | Dose $\times$ 2 |
| Azosemide | Dose $\times$ 2/3 |
| Ethacrynic acid | Dose $\times$ 4/5 |

**Supplemental Table 2. Effect of semaglutide vs. placebo on efficacy endpoints, stratified by diuretic use (no diuretics vs. any diuretic) pooled across the STEP-HFpEF and STEP-HFpEF-DM trials**

| **Endpoint** | **Parameter** | **No diuretics at baseline** | | **On diuretics (any kind) at baseline** | | **Interaction P-value** |
| --- | --- | --- | --- | --- | --- | --- |
|  |  | **Semaglutide (N=120)** | **Placebo (N=100)** | **Semaglutide (N=453)** | **Placebo (N=472)** |  |
| KCCQ-CSS, points | Baseline to 52-week change | 14.3 [10.9; 17.6] | 11.1 [7.4; 14.8] | 15.2 [13.5; 17.0] | 6.7 [5.0; 8.4] |  |
| KCCQ-CSS, points | Adjusted mean difference | 3.1 [-1.8; 8.1] | | 8.5 [6.1; 11.0] | | 0.055 |
| Body weight, % | Baseline to 52-week change | -11.4 [-12.7; -10.1] | -3.0 [-4.6; -1.5] | -11.4 [-12.1; -10.7] | -3.0 [-3.7; -2.3] |  |
| Body weight, % | Adjusted mean difference | -8.3 [-10.3; -6.3] | | -8.4 [-9.3; -7.4] | | 0.98 |
| 6MWD, m | Baseline to 52-week change | 18.2 [6.5; 30.0] | 0.3 [-13.0; 13.5] | 16.4 [10.2; 22.6] | -0.4 [-6.5; 5.6] |  |
| 6MWD, m | Adjusted mean difference | 18.0 [0.3; 35.6] | | 16.8 [8.1; 25.6] | | 0.91 |
| C-reactive protein | Baseline to 52-week ratio | 0.53 [0.44; 0.63] | 0.88 [0.71; 1.08] | 0.58 [0.53; 0.65] | 0.90 [0.82; 0.99] |  |
| C-reactive protein | Treatment ratio | 0.60 [0.46; 0.79] | | 0.65 [0.56; 0.75] | | 0.65 |
| Systolic BP, mmHg | Baseline to 52-week change | -1.7 [-4.6; 1.2] | -0.3 [-3.8; 3.2] | -5.4 [-6.9; -3.8] | -2.0 [-3.6; -0.5] |  |
| Systolic BP, mmHg | Adjusted mean difference | -1.4 [-5.9; 3.2] | | -3.3 [-5.6; -1.1] | | 0.44 |
| Waist circumference, cm | Baseline to 52-week change | -10.7 [-12.1; -9.2] | -3.3 [-5.1; -1.6] | -10.2 [-11.0; -9.4] | -2.5 [-3.3; -1.7] |  |
| Waist circumference, cm | Adjusted mean difference | -7.3 [-9.6; -5.0] | | -7.7 [-8.8; -6.6] | | 0.77 |
| KCCQ-OSS, points | Baseline to 52-week change | 14.6 [11.2; 17.9] | 10.7 [7.0; 14.4] | 15.0 [13.3; 16.8] | 6.8 [5.1; 8.5] |  |
| KCCQ-OSS, points | Adjusted mean difference | 3.9 [-1.1; 8.8] | | 8.2 [5.8; 10.6] | | 0.12 |
| NTproBNP | Baseline to 52-week ratio | 0.70 [0.60; 0.81] | 0.93 [0.78; 1.12] | 0.80 [0.74; 0.87] | 0.96 [0.88; 1.04] |  |
| NTproBNP | Treatment ratio | 0.75 [0.59; 0.95] | | 0.84 [0.75; 0.94] | | 0.39 |
| KCCQ-TSS, points | Baseline to 52-week change | 15.7 [12.2; 19.3] | 10.3 [6.3; 14.2] | 15.9 [14.1; 17.7] | 7.0 [5.2; 8.8] |  |
| KCCQ-TSS, points | Adjusted mean difference | 5.4 [0.2; 10.7] | | 8.9 [6.3; 11.5] | | 0.24 |
| KCCQ-PLS, points | Baseline to 52-week change | 13.3 [9.6; 17.1] | 11.9 [7.7; 16.1] | 14.5 [12.6; 16.5] | 6.1 [4.2; 8.0] |  |
| KCCQ-PLS, points | Adjusted mean difference | 1.5 [-4.1; 7.1] | | 8.4 [5.7; 11.1] | | 0.028 |
| KCCQ-SLS, points | Baseline to 52-week change | 15.2 [11.0; 19.4] | 10.5 [5.9; 15.2] | 13.5 [11.3; 15.7] | 6.4 [4.2; 8.5] |  |
| KCCQ-SLS, points | Adjusted mean difference | 4.7 [-1.6; 10.9] | | 7.1 [4.0; 10.2] | | 0.48 |
| KCCQ-QLS, points | Baseline to 52-week change | 15.6 [11.7; 19.4] | 10.9 [6.6; 15.2] | 15.4 [13.5; 17.4] | 7.6 [5.7; 9.6] |  |
| KCCQ-QLS, points | Adjusted mean difference | 4.6 [-1.1; 10.3] | | 7.8 [5.0; 10.6] | | 0.32 |

KCCQ = Kansas City Cardiomyopathy Questionnaire; CSS = Clinical Summary Score; 6MWD = 6-minute walk distance; BP = blood pressure; OSS = Overall Summary Score; NTproBNP = N-terminal pro-B-type natriuretic peptide; TSS = Total Symptom Score; PLS = Physical Limitation Score; SLS = Social Limitation Score; QLS = Quality of Life Score.

**Supplemental Table 3. Effect of semaglutide vs. placebo on efficacy endpoints, stratified by baseline loop diuretic use (no loop diuretic vs. any dose of loop diuretic) pooled across the STEP-HFpEF and STEP-HFpEF-DM trials**

| **Endpoint** | **Parameter** | **No loop diuretic at baseline** | | **Loop diuretic (any dose) at baseline** | | **Interaction P-value** |
| --- | --- | --- | --- | --- | --- | --- |
|  |  | **Semaglutide (N=229)** | **Placebo (N=214)** | **Semaglutide (N=344)** | **Placebo (N=358)** |  |
| KCCQ-CSS, points | Baseline to 52-week change | 15.0 [12.6; 17.4] | 10.3 [7.8; 12.8] | 15.1 [13.0; 17.1] | 5.8 [3.8; 7.7] |  |
| KCCQ-CSS, points | Adjusted mean difference | 4.7 [1.3; 8.2] | | 9.3 [6.5; 12.1] | | 0.042 |
| Body weight, % | Baseline to 52-week change | -11.6 [-12.5; -10.7] | -3.0 [-4.1; -2.0] | -11.2 [-12.0; -10.4] | -3.0 [-3.8; -2.2] |  |
| Body weight, % | Adjusted mean difference | -8.6 [-9.9; -7.2] | | -8.2 [-9.3; -7.1] | | 0.70 |
| 6MWD, m | Baseline to 52-week change | 20.2 [11.7; 28.7] | 1.0 [-8.0; 9.9] | 14.5 [7.3; 21.7] | -1.1 [-8.2; 6.0] |  |
| 6MWD, m | Adjusted mean difference | 19.3 [7.0; 31.5] | | 15.6 [5.5; 25.7] | | 0.65 |
| C-reactive protein | Baseline to 52-week ratio | 0.57 [0.50; 0.65] | 0.86 [0.75; 0.99] | 0.57 [0.51; 0.65] | 0.92 [0.83; 1.02] |  |
| C-reactive protein | Treatment ratio | 0.67 [0.55; 0.80] | | 0.62 [0.53; 0.73] | | 0.58 |
| Systolic BP, mmHg | Baseline to 52-week change | -2.8 [-4.9; -0.7] | -1.1 [-3.5; 1.2] | -5.8 [-7.6; -4.0] | -2.1 [-3.9; -0.3] |  |
| Systolic BP, mmHg | Adjusted mean difference | -1.7 [-4.8; 1.4] | | -3.7 [-6.2; -1.2] | | 0.32 |
| Waist circumference, cm | Baseline to 52-week change | -10.5 [-11.6; -9.5] | -3.1 [-4.3; -1.9] | -10.1 [-11.0; -9.1] | -2.4 [-3.3; -1.4] |  |
| Waist circumference, cm | Adjusted mean difference | -7.5 [-9.1; -5.9] | | -7.7 [-9.0; -6.4] | | 0.81 |
| KCCQ-OSS, points | Baseline to 52-week change | 15.1 [12.6; 17.5] | 10.0 [7.5; 12.5] | 14.9 [12.8; 16.9] | 6.0 [4.0; 7.9] |  |
| KCCQ-OSS, points | Adjusted mean difference | 5.0 [1.6; 8.5] | | 8.9 [6.1; 11.7] | | 0.086 |
| NTproBNP | Baseline to 52-week ratio | 0.71 [0.64; 0.80] | 0.96 [0.85; 1.09] | 0.83 [0.75; 0.91] | 0.95 [0.86; 1.04] |  |
| NTproBNP | Treatment ratio | 0.74 [0.63; 0.88] | | 0.87 [0.76; 1.00] | | 0.14 |
| KCCQ-TSS, points | Baseline to 52-week change | 16.0 [13.4; 18.5] | 9.2 [6.6; 11.9] | 15.8 [13.7; 17.9] | 6.5 [4.5; 8.6] |  |
| KCCQ-TSS, points | Adjusted mean difference | 6.7 [3.0; 10.4] | | 9.3 [6.3; 12.2] | | 0.28 |
| KCCQ-PLS, points | Baseline to 52-week change | 14.4 [11.7; 17.1] | 11.6 [8.8; 14.4] | 14.2 [12.0; 16.4] | 4.4 [2.2; 6.6] |  |
| KCCQ-PLS, points | Adjusted mean difference | 2.8 [-1.1; 6.6] | | 9.8 [6.7; 12.9] | | 0.005 |
| KCCQ-SLS, points | Baseline to 52-week change | 15.1 [12.0; 18.1] | 10.7 [7.5; 13.8] | 13.1 [10.5; 15.6] | 4.9 [2.5; 7.4] |  |
| KCCQ-SLS, points | Adjusted mean difference | 4.4 [0.1; 8.7] | | 8.1 [4.6; 11.7] | | 0.18 |
| KCCQ-QLS, points | Baseline to 52-week change | 15.7 [12.9; 18.4] | 10.2 [7.3; 13.1] | 15.3 [13.0; 17.6] | 7.0 [4.7; 9.2] |  |
| KCCQ-QLS, points | Adjusted mean difference | 5.5 [1.5; 9.5] | | 8.3 [5.1; 11.5] | | 0.27 |

KCCQ = Kansas City Cardiomyopathy Questionnaire; CSS = Clinical Summary Score; 6MWD = 6-minute walk distance; BP = blood pressure; OSS = Overall Summary Score; NTproBNP = N-terminal pro-B-type natriuretic peptide; TSS = Total Symptom Score; PLS = Physical Limitation Score; SLS = Social Limitation Score; QLS = Quality of Life Score.

**Supplemental Table 4. Effects of semaglutide vs. placebo on loop diuretic dose changes from baseline to 20 weeks (A), and baseline to 36 weeks (B), pooled across the STEP-HFpEF and STEP-HFpEF-DM trials**

**A.**

| **Loop Diuretic Change** | **Semaglutide 2.4 mg** | **Placebo** | **OR (95% CI; Semaglutide 2.4 mg vs. placebo)** | **p-value** |
| --- | --- | --- | --- | --- |
|  |  |  |  |  |
| Dose Decrease | 44 (7.8) | 38 (6.8) | 1.33 (0.83, 2.14) | 0.234 |
|  |  |  |  |  |
| No Change | 490 (87.3) | 464 (83.6) | 1.29 (0.91, 1.82) | 0.146 |
|  |  |  |  |  |
| Dose Increase | 27 (4.8) | 53 (9.5) | 0.48 (0.30, 0.78) | 0.003 |
|  |  |  |  |  |

**B.**

| **Loop Diuretic Change** | **Semaglutide 2.4 mg** | **Placebo** | **OR (95% CI; Semaglutide 2.4 mg vs. placebo)** | **p-value** |
| --- | --- | --- | --- | --- |
|  |  |  |  |  |
| Dose Decrease | 61 (11.1) | 37 (6.9) | 1.96 (1.25, 3.07) | 0.003 |
|  |  |  |  |  |
| No Change | 460 (83.9) | 434 (80.8) | 1.18 (0.85, 1.63) | 0.317 |
|  |  |  |  |  |
| Dose Increase | 27 (4.9) | 66 (12.3) | 0.38 (0.24, 0.60) | <0.001 |
|  |  |  |  |  |

**Supplemental Figure 1. Histogram of baseline loop diuretic doses pooled across the STEP-HFpEF and STEP-HFpEF-DM trials**
